# Effects of temperature and phytoplankton community composition on subitaneous and resting egg production rates of *Acartia omorii* in Tokyo Bay

**DOI:** 10.1038/s41598-021-86976-8

**Published:** 2021-04-12

**Authors:** Ayumi Tsunashima, Hiroshi Itoh, Toshiya Katano

**Affiliations:** 1 Graduate School of Marine Science and Technology, Tokyo University of Marine Science and Technology, Konan 4-5-7, Minato, Tokyo, 108-8477 Japan; 2 Suidosha Co. Ltd, 8-11-11 Ikuta, Kawasaki, Kanagawa 214-0038 Japan

**Keywords:** Marine biology, Community ecology, Population dynamics

## Abstract

To clarify the effects of temperature and phytoplankton community composition on *Acartia omorii* (Copepoda: Calanoida) egg production, its abundance and egg production rates were investigated from 2016 to 2018 in Tokyo Bay, Japan. Abundance was high from March to May (> 3.0 × 10^4^ individuals m^−3^) and low or undetected from late June to December (≤ 0.4 × 10^4^ individuals m^−3^). In 2018, most eggs were subitaneous until April; diapause eggs appeared in May when the water temperature exceeded 20 °C. The weight-specific egg production rate (SEPR, C_egg_ C_female_^−1^ day^−1^) had two peaks. In the first peak in January, > 90% of eggs were subitaneous; in contrast, in the second peak in May, 60% of eggs were unhatched, including diapause eggs. The first peak of subitaneous eggs may contribute to planktonic population development from March to May. In contrast the second peak of diapause eggs probably enhances their recurrence in the next winter. Multiple regression analysis revealed that subitaneous SEPR showed a negative response, whereas diapause SEPR showed a positive response to temperature. Subitaneous SEPR positively correlated with the proportion of small diatoms in phytoplankton carbon biomass, whereas unhatched SEPR positively correlated with the proportion of inedible preys in large diatoms and dinoflagellates. Edible diatoms may induce subitaneous egg production, whereas low-food availability may induce diapause egg production. These results suggest that phytoplankton composition and water temperature have strong impacts on the dynamics of *A. omorii* via egg production.

## Introduction

Planktonic calanoid copepods are important in the coastal ecosystem; they are phytoplankton and microzooplankton consumers and prey of fish larvae^1,2^. *Acartia* is a representative genus in coastal waters worldwide. Its egg production has been intensively investigated to clarify its population dynamics^3,4^. Many *Acartia* species produce diapause and subitaneous eggs^5,6^. Subitaneous eggs hatch immediately after spawning; however, some remain in a quiescent stage under unfavorable environmental conditions for development or hatching^7^. Diapause eggs have a refractory phase during which they do not resume development, even in favorable environments^8^. Apart from these two egg types, *Acartia tonsa*, *A. japonica,* and *A. steueri* also produce delayed-hatching eggs that hatch over a wide time span^4,6,9,10^. Delayed-hatching eggs do not hatch faster than subitaneous eggs, and the refractory period of delayed-hatching eggs is shorter than that of diapause eggs^11^. Takayama and Toda^4^ investigated seasonal variation in egg production in *A. japonica* and reported the timing and nature of switching between the three egg types. Nevertheless, there is little information on the effect of seasonal variation in the egg types produced by individual female copepods in the field.

*A. omorii* Bradford had been attributed to *A. clausi* Giesbrecht in the northwest Pacific and distinguished from *A. clausi*, which is the most common calanoid copepod in inlet waters^12^. *A. omorii* is present from northern Hokkaido (41°N) to southern Kyushu (32°N)^13^. It favors colder environments; its upper critical thermal level is 20–25 °C. Therefore, in cold waters, such as Onagawa Bay (38.4°N), planktonic population occurs throughout the year; its abundance peaks during summer and autumn^14^. In warmer waters, such as Tokyo Bay (35.3°–35.7°N) and the Seto Inland Sea (34.3°–34.7°N), planktonic population disappears from the water column due to high water temperature during summer^15–18^.

The in situ egg production rate (EPR) (eggs female^−1^ day^−1^) of *A. omorii* has been measured in Onagawa Bay^3,19^. Uye^3^ studied the effects of water temperature, chlorophyll *a* concentration, and differences in female carbon content between summer and winter on egg production via laboratory experiments and derived a simple model equation describing EPR using these three factors. This model equation is almost consistent with seasonal variation in in situ EPR in Onagawa Bay, where few diapause eggs are produced^5,7^. In contrast, in Tokyo Bay and the Seto Inland Sea, *A. omorii* produces diapause eggs not morphologically different from subitaneous eggs^5,15,18,20^. According to Uye^5^, diapause egg production is triggered by longer day length (> 14 h) and higher water temperature (> 18 °C). However, seasonal changes in EPR and the effects of environmental conditions on EPR remain unelucidated.

Egg production by *A. omorii* is considered to be saturated at low chlorophyll *a* concentrations (1–2 µg L^−1^)^3,19^. However, several studies suggest that prey organisms affect EPR. Ingestion of diatom species by adult females causes anomalous eggs and naupliar development, low EPR, and low hatching success rate^21^. *A. omorii* rejected the ingestion of several red tide flagellates due to their toxicity^22^. The prey’s nutritional quality is also an important factor affecting EPR. The fertility of *A. omorii* was improved by feeding them dinoflagellates, which contain more polyunsaturated fatty acids than diatoms, suggesting that diatoms and dinoflagellates have different nutritional effects on egg production^23^. Because *Acartia* species have little energy reserve^24^, food availability readily affects their egg production^25^. Phytoplankton composition in their habitat may be important for egg production. However, the effect of phytoplankton composition on in situ EPR of *A. omorii* remains uninvestigated.

Tokyo Bay is highly eutrophic and productive^26,27^, with *A. omorii* as the dominant copepod species from winter to early summer^16,18,28^, whereas *Oithona davisae* is dominant in summer^18^. Seasonal changes in egg production and shift from subitaneous to diapause eggs are not well-documented in natural environments. *A. omorii* probably forms diapause eggs in early summer. Recently, Tachibana et al.^29^ reported that the decadal mean abundance of *A. omorii* for the 2000s decreased by 58.7% from the 1980s. The shift in production from subitaneous to resting eggs was due to changes in abiotic factors, such as water temperature. Besides the water temperature, phytoplankton composition has also drastically changed over the past few decades^30^. Since 2000, Euglenophyceae appear in winter, with a longer period of appearance. Additionally, large dinoflagellates, such as *Ceratium fusus* and *C. furca*, have become dominant, regardless of the season^30,31^. Such shifts in phytoplankton composition might affect egg production by *A. omorii*.

To better understanding *A. omorii*’s life cycle, we investigated the seasonal dynamics of phytoplankton population with abiotic and biotic environmental variables at two stations in Tokyo Bay for 2 years. EPR was also investigated for 17 months. Our specific aims were to describe seasonal changes in egg production by *A. omorii* in a bay where diapause eggs are produced and determine the controlling mechanism of egg production.

## Materials and methods

### Field monitoring

**Figure 1:**
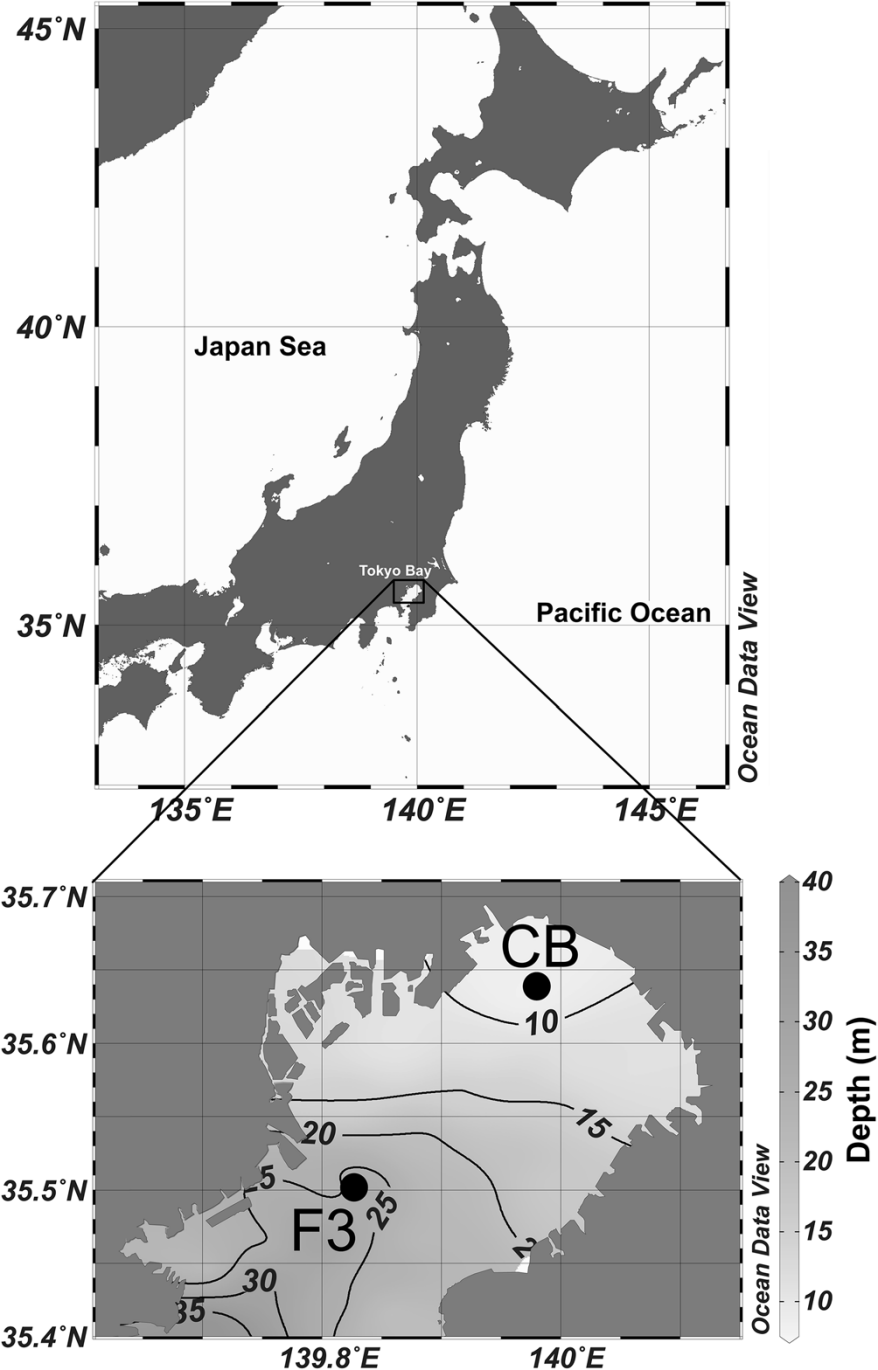
Location of the sampling station: St. F3 and St. CB in Tokyo Bay (St. F3: 35° 30′ 30″N, 139° 50′ 00″E) (St. CB: 35° 38′ 00″N, 139° 58′ 00″E). Lines indicate bathymetric contours. Maps were drawn using ODV version 5.0.0 (Schlitzer, Reiner, Ocean Data View, 2020, https://odv.awi.de/). Bathymetric data were obtained from Ministry of Land, Infrastructure, Transport and Tourism (https://www1.kaiho.mlit.go.jp/KAN3/kaisyo/tokyo_kankyo/htm/kakodata.html).

Field sampling was conducted monthly from January 2016 to June 2018 at two stations (station F3, 27 m depth and station CB, 8 m depth) in Tokyo Bay (Fig. 1) using a Hiyodori training boat of Tokyo University of Marine Science and Technology. Water temperature, salinity, and chlorophyll fluorescence were measured using CTD (AAQ1183-PRO, JFE–Advantech, Nishinomiya, Japan, from May 16, 2017 or AAQ177, JFE–Advantech, after June 28, 2018) at each station. Water samples were vertically collected with a 2-L Van Dorn water sampler (Rigosha, Tokyo, Japan) and shipped to the laboratory on ice. Zooplankton samples were collected by vertical tows from 2 m above the surface bottom using a plankton net (mouth diameter, 0.45 m; mesh opening, 100 µm) equipped with a flowmeter (Rigosha). Samples were immediately fixed and preserved in 5% formalin seawater solution. To determine the carbon biomass of phytoplankton, seawater samples were fixed with formalin solution to a final concentration of 1%.

To determine chlorophyll *a* concentration, 50 mL of water was filtered through a glass-fiber filter (GF/F, Whatman, GE Healthcare, IL, USA). Chlorophyll *a* concentration was determined using the fluorometric method^32^ with a fluorometer (Model 10-AU, Turner Designs, San Jose, CA, USA) after extraction with *N*,*N*-dimethylformamide.

Adults and copepodites of *A. omorii* from split subsamples of the original sample were counted under a stereomicroscope (SZ61, Olympus, Tokyo, Japan); there were usually > 200 individuals in each sample except when abundance was low. The numbers of males and females from copepodite stage IV to adults were counted. The count data were converted to abundance per unit volume (individuals m^−3^).

Phytoplankton cells were counted under an optical microscope (CX22LED, Olympus) using a counting plate (MPC-200, Matsunami Glass, Tokyo, Japan); there were usually > 400 cells for each species. For the minor species, we counted cells in 200 µL of the sample. The biovolume per phytoplankton cell (*V*, µm^3^) was calculated from the minimum and maximum diameters of the cell measured using an ocular micrometer according to the following cell volume conversion equations for five cell types^33^:

- Elliptical cylinder: *V* = 3/20 π *a*^2^ *b*,
- Elliptical cone, spindle, and partial spindle: *V* = 1/20 π *a*^2^ *b*,
- Flat ellipsoid: *V* = 1/10 π *a*^2^ *b*,
- Ellipsoid: *V* = 1/6 π *a*^2^ *b*,
- Rectangular parallelepiped: *V* = 3/5 *a*^2^ *b*,

where *a* is the minimum diameter and *b* is the maximum diameter. Cell volume was then converted to carbon content per cell (*C*: pg cell^−1^) using Strathmann's^34^ equations:

- Diatoms: log *C* = −0.442+0.758 log *V*,
- Other phytoplankton: log *C* = −0.460+0.866 log *V*.

The carbon content per cell was multiplied by each plankton’s cell density and converted to carbon biomass (µg C L^−1^). Phytoplankton was classified into six groups according to carbon biomass: small and large diatoms, small and large dinoflagellates, nanoflagellates, and others, including dictyochophytes. The cutoff between small and large was set at 100 µm, assuming that the edible size range for *A. omorii* was 5–100 µm^35,36^. Generally, copepods can feed on particles < 20% the length of their prosomes and can swallow particles 3–10% the length of their prosomes^36^. Therefore, small diatoms and dinoflagellates are assumed to be edible by adult females of *A. omorii*, which have a prosome length of > 600 µm^17^.

### EPR experiment

EPR was measured 17 times at station F3 and 15 times at station CB from February 2017 to June 2018. Adult females were collected by vertical tows from 2 m above the surface bottom using a plankton net (diameter 0.45 m, mesh opening 200 µm) with a specially designed 2-L plastic bottle at the cod end not to damage copepod individuals. Immediately after collection, adult *A. omorii* female from the net sample was placed in 60-mL polyethylene terephthalate screw vial (diameter 40 mm, length 73.5 mm) using a stereo microscope on board. Fifteen EPR measurements were performed for each station. Each vial was filled with approximately 40 mL of seawater collected from a 5-m depth using a Van Dorn water sampler and filtered through a hand net (mesh opening 10 µm). To prevent predation by adult females on their own eggs, a chamber (diameter 26 mm, height 70 mm) made by stretching a 100-µm net over an acrylic pipe was installed to each vial so that eggs approximately 70 µm in diameter^14,20^ would fall to the bottom. After removing from the boat, the vials were immediately placed in an incubator maintained at an in situ temperature of 5-m depth. The light–dark cycle was adjusted for each sampling day. Twenty-four hours after on-board sorting, adult females were removed from each vial. At that time, the survival of each individual was confirmed; EPR for dead individuals was not measured. Prosome length was measured for all tested individuals using an imaging software (DP2-SAL, Olympus) with a CMOS camera (DP27, Olympus) equipped with an inverted microscope (CKX53, Olympus).

The eggs left in the containers were placed in 12-well plates and further incubated under the same temperature and light conditions as mentioned above. Up to March 2018, eggs hatching after 48–96 h of incubation were classified as subitaneous eggs; those that remained unhatched were classified as unhatched eggs. After April 2018, unhatched eggs were confirmed by culturing them in filtered seawater at in situ temperatures for the first 7 days and then continuing incubation for approximately 2 weeks while gradually lowering the temperature. The temperature during the additional 2 weeks was reduced by 2 °C every 1–3 days and finally to 10 °C, which was close to the winter water temperature. During the first 7 days of incubation, eggs that hatched as the water temperature decreased were classified as quiescent, eggs that rotted were non-viable, and eggs that neither hatched nor rotted (attached bacteria or egg exterior is not dark)^37^ were diapause eggs. From April to June 2018, the egg type composition was recorded for each individual to determine the temporal shift in spawned eggs by individuals. Data from individuals that did not lay eggs were excluded because they might not have been fertilized. EPR (eggs female^−1^ day^−1^) and its mean value on each sampling day were calculated according to the number of eggs produced. The production rate of unhatched and subitaneous eggs were estimated by the hatching rate. To evaluate egg production rate without the effect of seasonal changes in body size, the weight-specific egg production rate (SEPR) (C_egg_ C_female_^−1^ day^−1^) was calculated using egg carbon content (µg) and female carbon weight (C_female_, µg) of an individual estimated from the length–weight relationship^38^:

$${\text{C}}_{{{\text{female}}}} = {\text{ L}}_{{\text{p}}}^{{{3}.0{8}}} \times {1}0^{{ - {8}.{51}}}$$

where L_p_ is prosome length (µm). Because the measured egg size of *A. omorii* was 72.1–74.5 µm, which is similar to that reported by Uye^14^, and changed little according to season, we used the egg carbon content value of 0.025 µg C egg^−1^ measured by Uye^3,14^. The population egg production rate (PEPR) (eggs m^−3^ day^−1^) was calculated from EPR (eggs female^−1^ day^−1^), adult female abundance, and female–male ratio.

### Statistical analysis

To examine whether EPR differed between the two stations, one-way analysis of variance was performed and multiple comparisons were conducted using the Tukey's post hoc test. Relationships were examined between weight-specific EPR (subitaneous and unhatched eggs, including resting eggs) and the following environmental factors: water temperature (surface, bottom, and incubation), salinity (surface and bottom), chlorophyll *a* concentration (surface, peak, and bottom), phytoplankton carbon biomass (small and large diatoms, small and large dinoflagellates, nanoflagellates, and others), and phytoplankton carbon biomass ratio (same as above). Each environmental factor was standardized to a mean of 0 and a variance of 1 and examined for multicollinearity based on the correlation matrix and variance inflation factors (to perform multiple regression analysis with the noncollinearity environmental variable as the dependent variable). The model equation with minimum Akaike's information criterion was chosen as the best.

## Results

### Environmental variables

**Figure 2:**
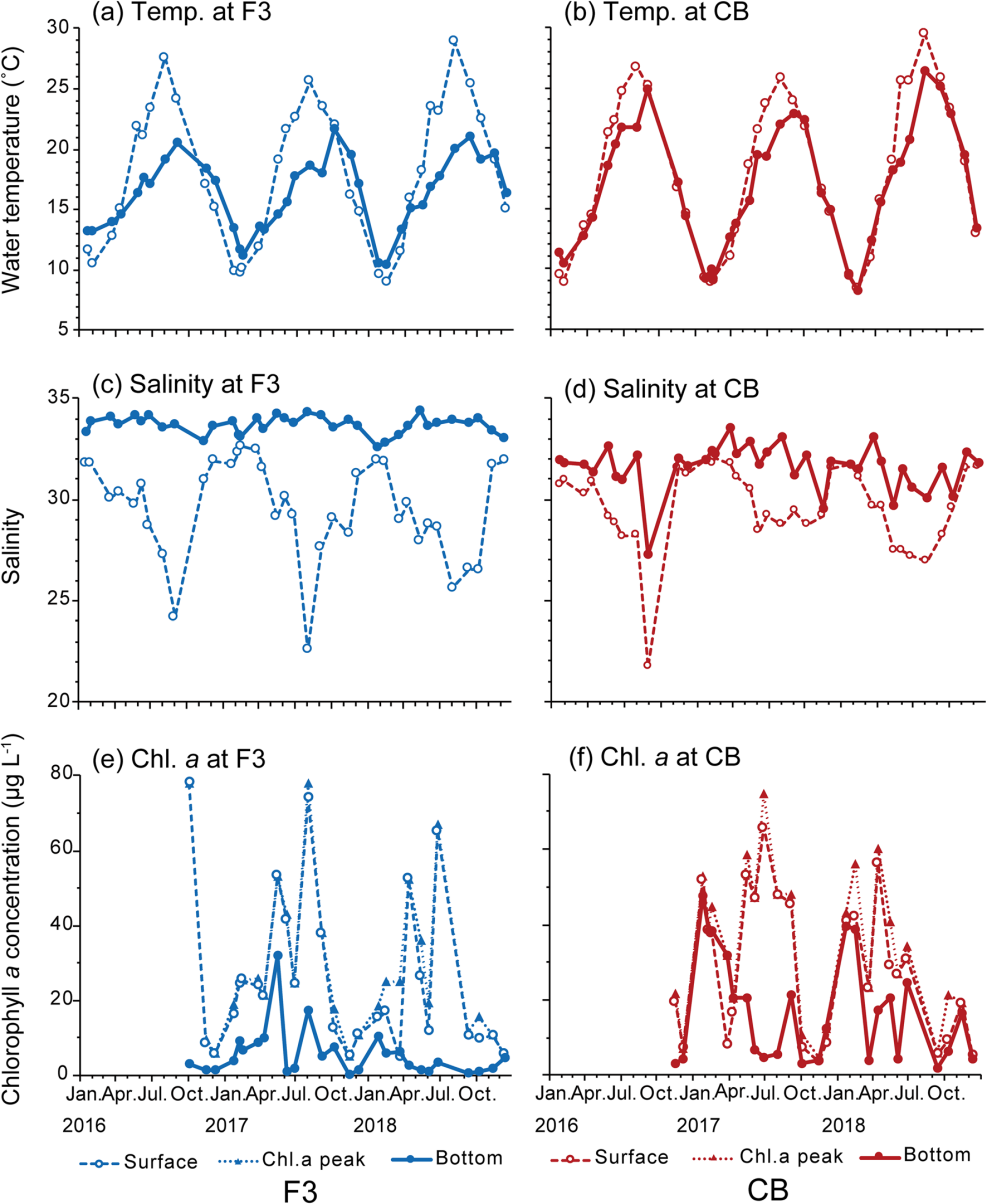
Seasonal changes in water temperature (**a**), salinity (**b**) and chlorophyll *a* concentration (Chl.) at St. F3 (**c**), St. CB (**d**). (**a**), (**b**): from January 21, 2016 through June 26, 2018. (**c**), (**d**): from October 5, 2016 through June 26, 2018.

Water temperatures were 8.9–27.6 °C and 8.2°–26.7 °C at stations F3 and CB, respectively (Fig. 2a). The temperature was lowest in February 2018 and highest at the surface at both stations in August 2016. The surface temperature exceeded 20 °C from May to September or October at both stations. Because the water column was shallower at station CB than at station F3, the bottom temperature was higher at station CB than at station F3 in summer (May–September) and lower in winter (November–March).

Salinity was 22.6–34.4 and 21.7–33.5 at stations F3 and CB, respectively (Fig. 2b). Salinity was always lower at station CB than at station F3. From April or May to September or October, surface salinity was < 30 due to rain. Surface salinity was the lowest in August 2017 at station F3 and in September 2016 at station CB. High salinity was recorded near the bottom in May 2018 at station F3 and in March 2017 at station CB.

Chlorophyll *a* concentration was measured from October 2016 (Fig. 2c,d). At both stations, chlorophyll *a* concentration was high from April to August, with highest values of 77.9 µg L^−1^ in October 2016 at station F3 and 75.0 µg L^−1^ in late June 2017 at station CB. At station F3, chlorophyll *a* concentration became low in winter; however, at station CB, high concentrations (50 µg L^−1^) were detected even in January and February.

### Seasonal variation in abundance

**Figure 3:**
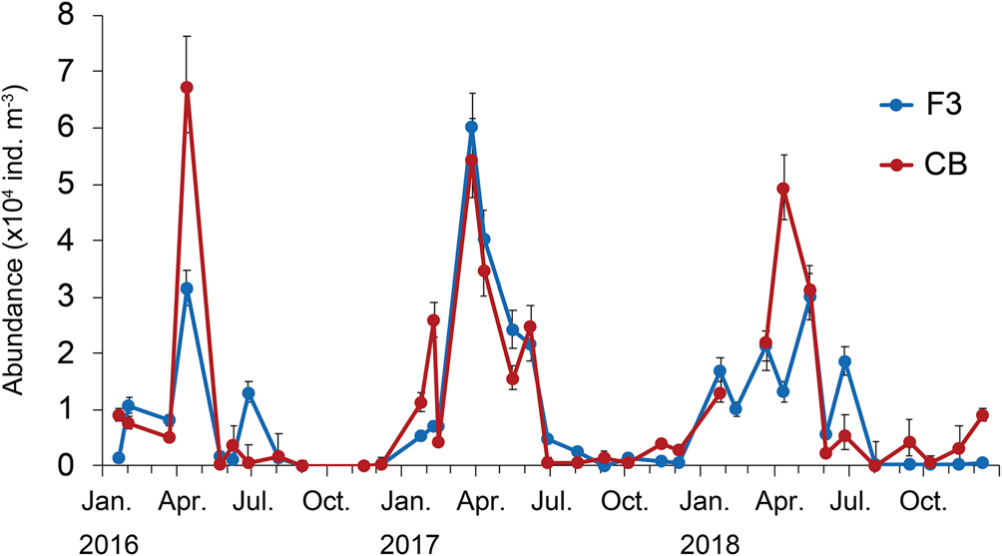
Seasonal changes in *Acartia omorii* abundance at St. F3 and St. CB from January 21, 2016 through June 26, 2017.

The abundance of *A. omorii* was high from March to May in 2016–2018 (Fig. 3). The maximum abundance reached > 6.0 × 10^4^ individuals m^−3^ at both stations. The population drastically declined from June, when the surface water temperature increased to approximately 20 °C (Fig. 2a). *A. omorii* disappeared from the water column from September to November 2016 and appeared again in December 2016. Overall, the abundance was low (≤ 0.40 × 10^4^ individuals m^−3^) between September and December in both years (2016 and 2017).

**Figure 4:**
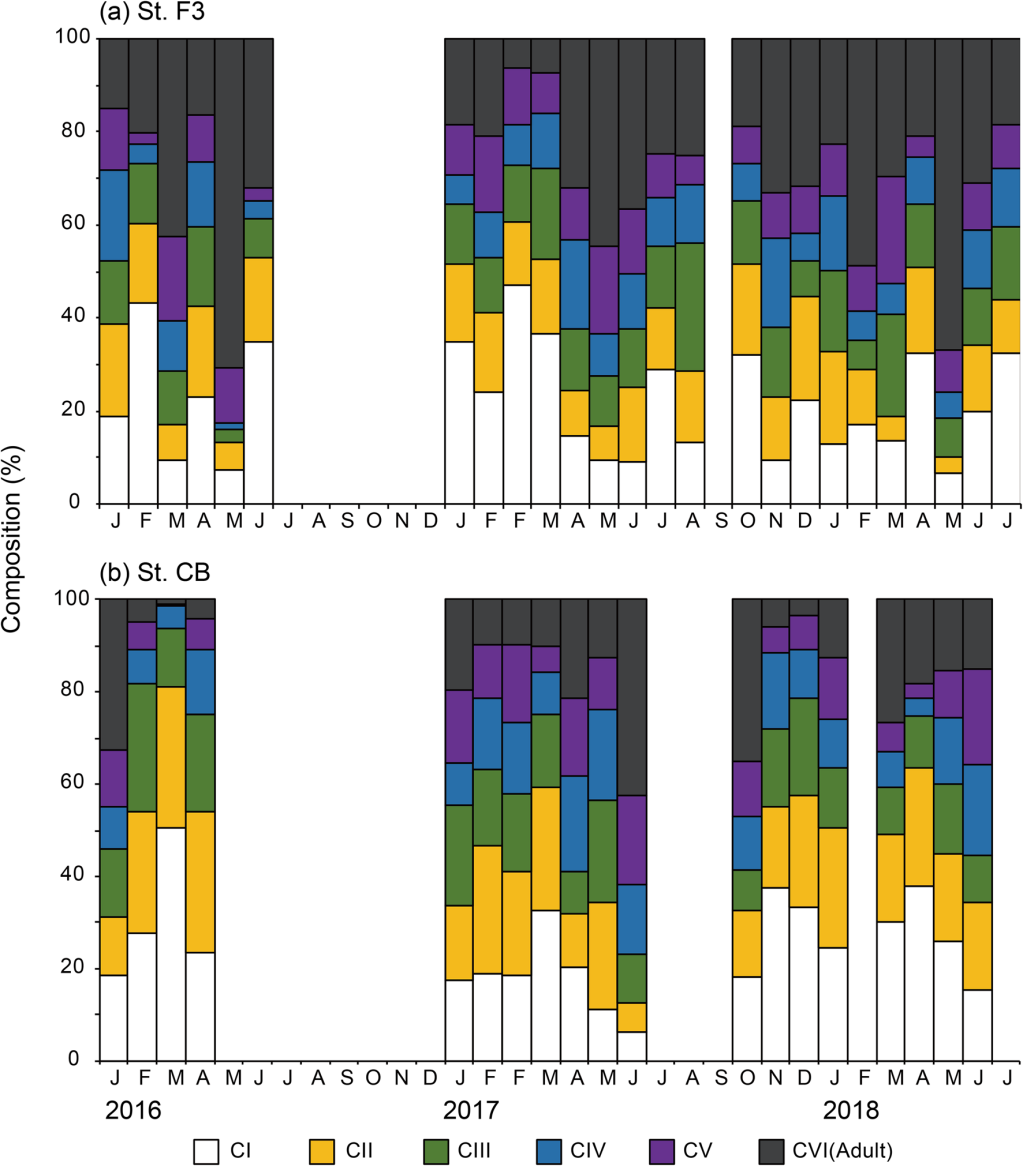
Seasonal changes in the composition of *Acartia omorii* stage-specific (CI–CVI) abundance at St. F3 (**a**) and St. CB (**b**) from January 21, 2016 through June 26, 2018. In 2017, the first F is on February 6, the second F is on February 14.

The percentage of stage I (CI)–III (CIII) younger copepodites frequently exceeded 60% between February and April at both stations when the *Acartia* population developed (Figs. 3, 4). After the peak period of abundance, the percentage of stage V (CV) to VI (CVI) copepodites peaked at 62–82% in May or June when the abundance declined, indicating a decline in the new recruitment. Interestingly, early stages again accounted for > 40% of the total population in June, probably due to the much higher number of subitaneous eggs produced by mature *Acartia* in the peak period. However, the abundance decreased to 5 × 10^2^–5 × 10^3^ individuals m^−3^ apparently due to decreased contribution of these early stages to population growth.

### Seasonal variation in body size and sex ratio

**Figure 5:**
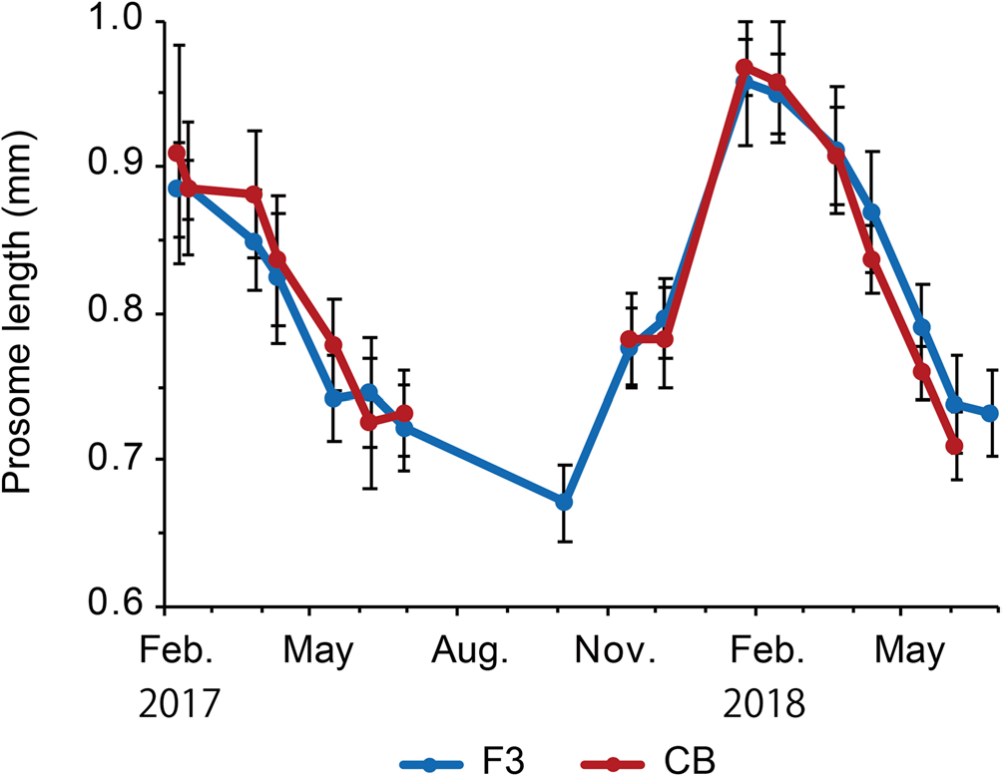
Seasonal changes in prosome length of adult female by used for egg production experiments of *Acartia omorii* at St. F3 and St. CB from February 2017 through June 2018. Error bars denote ± SD.

The prosome length of adult females ranged from 0.67 ± 0.025 mm at station F3 on October 5, 2017, to 0.97 ± 0.018 mm at station CB on January 25, 2018 (Fig. 5), showing an opposite pattern to seasonal changes in water temperature (Fig. 2). Body carbon weight, derived from prosome length, was 1.6 ± 0.19–4.7 ± 0.57 µg at station F3 and 1.9 ± 0.20–4.9 ± 0.29 µg at station CB. Body carbon weight decreased with increasing water temperature, suggesting a significant negative correlation with surface water temperature (*n* = 372, *r* =  − 0.83, *p* < 0.01).

**Figure 6:**
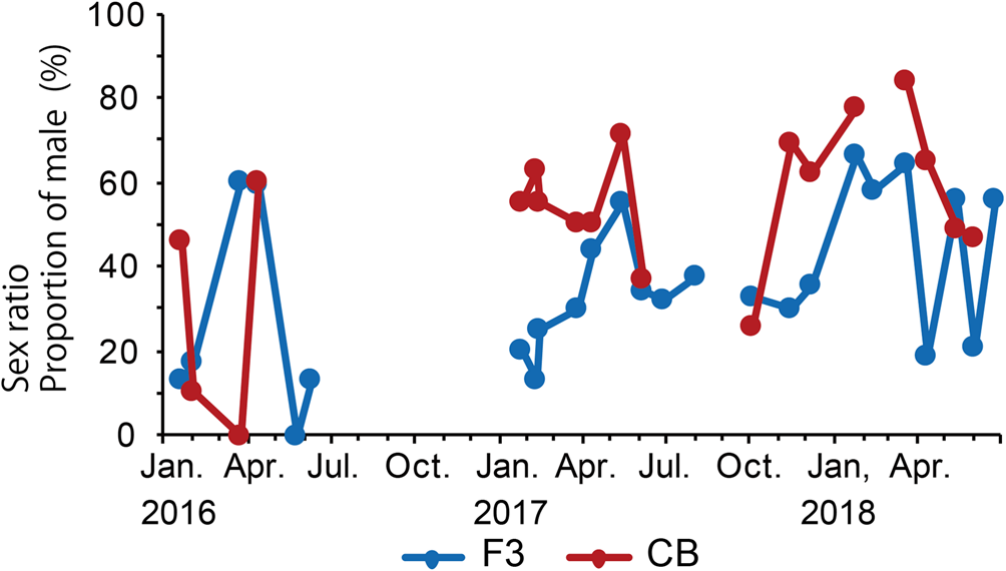
Seasonal variation in sex composition in adult male of *Acartia omorii* at St. F3 and St. CB from January 21, 2016 through June 26, 2018.

The proportion of males was high (55–84%) from March to May in both years at both stations, and then the proportion became low (0–47%) from May to June at both stations. Similar results were also obtained in Liang and Uye^17^. In general, male copepods have shorter life spans and experience higher mortality than female^39^. Such higher mortality may relate to the decline of male after May^17^ (Fig. 6).

### Seasonal changes in EPR

**Figure 7:**
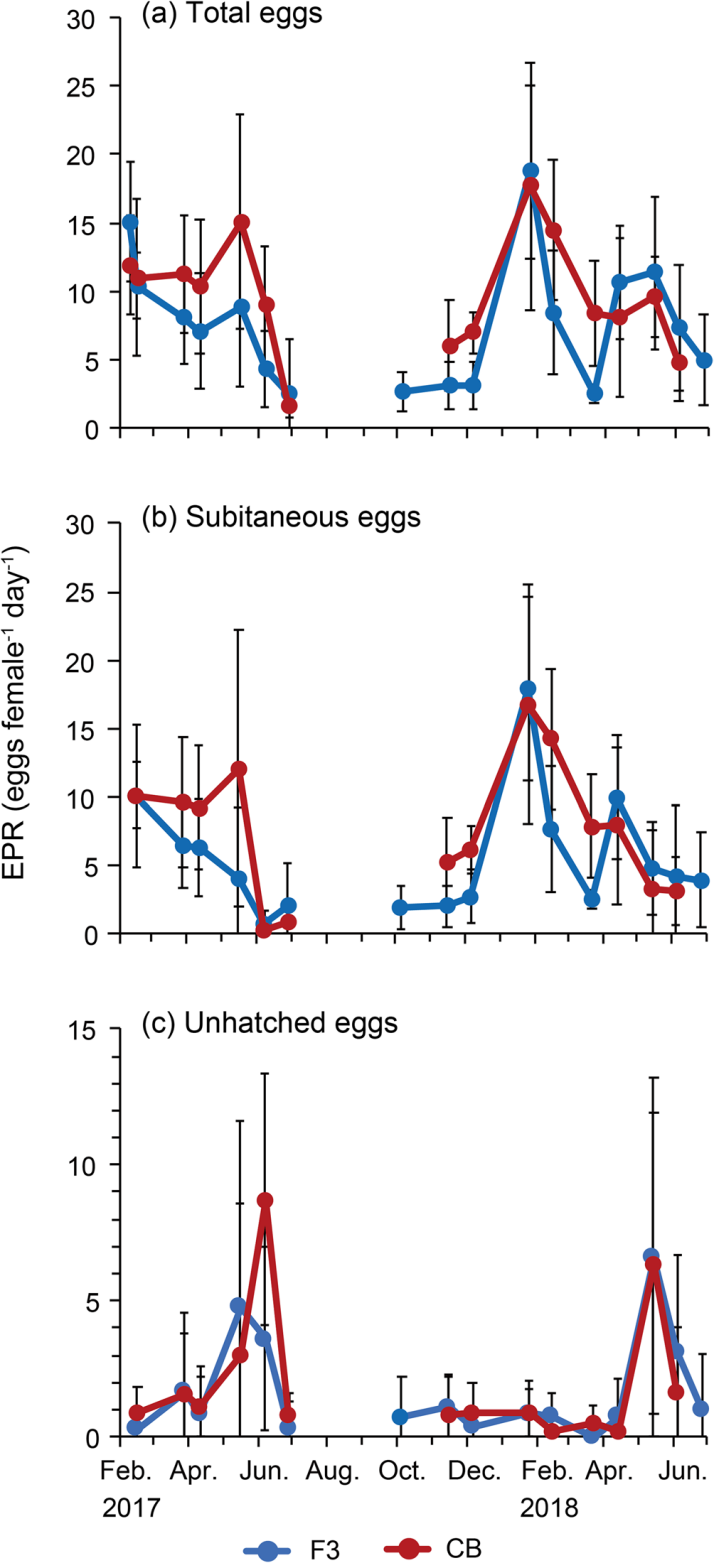
Seasonal changes in (**a**) total egg production rate (EPR), (**b**) Subitaneous EPR and (**c**) unhatched EPR of *Acartia omorii* at St. F3 and St. CB from February 2017 through June 2018. Error bars denote ± SD. *: significant difference between St. F3 and St. CB (Turkey's post-hoc test: *p* < 0.01).

Total EPR, including subitaneous and unhatched eggs, were 2.4 ± 4.0–18.7 ± 6.3 and 1.6 ± 0.9–17.7 ± 9.0 eggs female^−1^ day^−1^ at stations F3 and CB, respectively (Fig. 7a). EPR was high (> 10 eggs female^−1^ day^−1^) in winter (January and February) at both stations and declined to < 5.0 eggs female^−1^ day^−1^ toward the end of June in both years when the abundance decreased. The lowest value was observed on June 28, 2017. In April and May, EPR increased by 1.5–8.2 eggs female^−1^ day^−1^ from the previous month. During the study period, significantly higher fecundity was observed at station CB on June 7, 2017, December 6, 2018, and February 14, 2018 (Tukey–Kramer test, *p* < 0.01) (Fig. 7a, asterisk).

Subitaneous EPR fluctuated from 0.2 to 17.9 eggs female^−1^ day^−1^ (Fig. 7b). Seasonal changes in patterns were similar to those of total EPR. However, in May 16, 2017 and June 4, 2018, the rate drastically dropped to 0.1–2.3 eggs female^−1^ day^−1^ at station F3 when compared with the reduction in total EPR. Unhatched EPR was low (0–1.7 eggs female^−1^ day^−1^) from October to February. From May to June, with a decrease in subitaneous EPR (Fig. 7b), unhatched EPR became high (4.8–8.7 eggs female^−1^ day^−1^) (Fig. 7c). Unhatched eggs appeared just before the decreases in population density observed in 2017 and 2018 (Fig. 3), suggesting that unhatched eggs are produced to escape from high temperatures.

### Seasonal changes in SEPR

**Figure 8:**
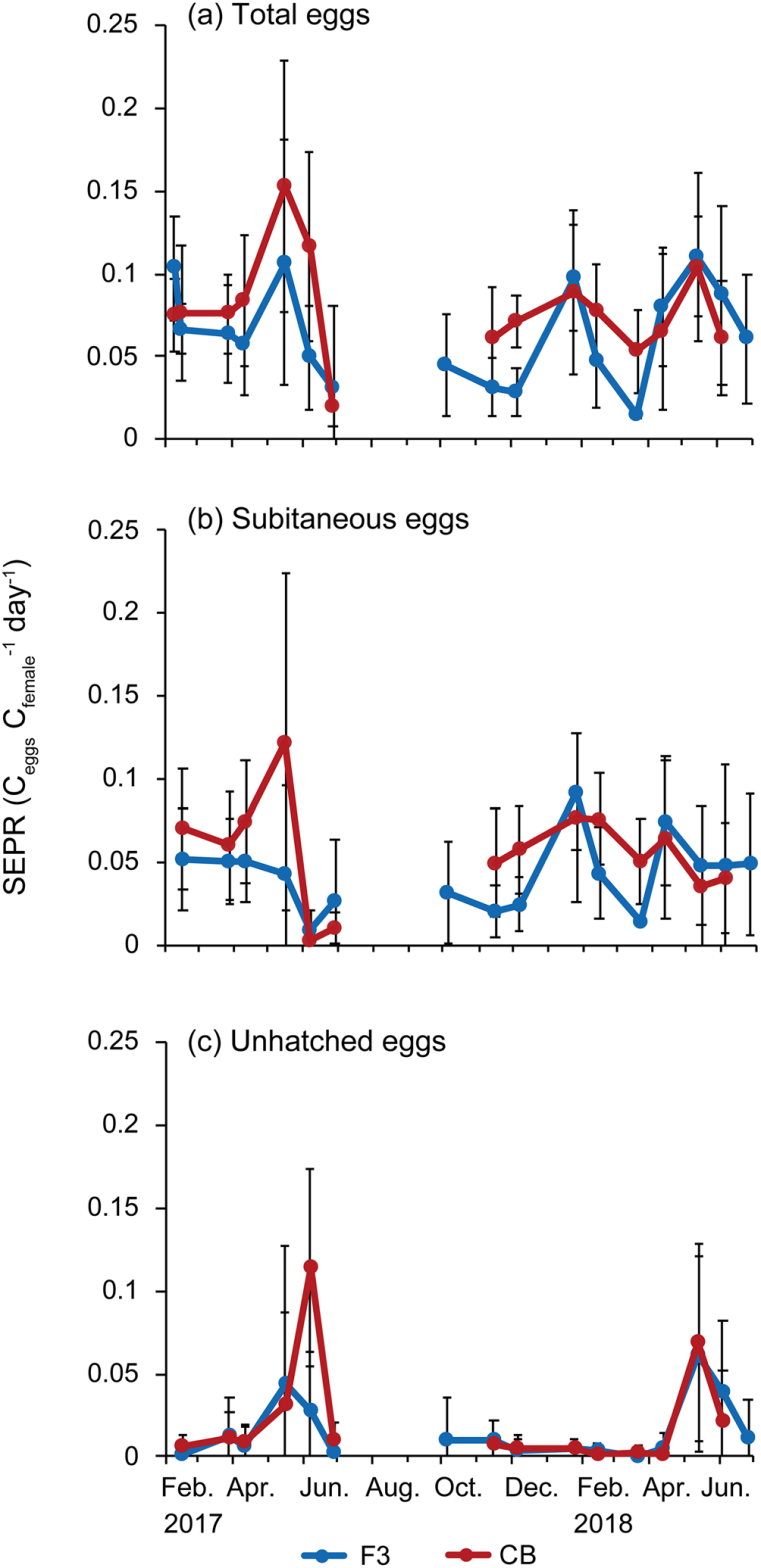
Seasonal changes in (**a**) total weight-specific egg production rate (SEPR), (**b**) Subitaneous SEPR and (**c**) Unhatched SEPR of *Acartia omorii* at St. F3 and St. CB from February 2017 through June 2018. Error bars denote ± SD.

Total SEPR fluctuated over a similar range at the two stations from 0.015 ± 0.0015 to 0.11 ± 0.051 C_egg_ C_female_^−1^ day^−1^ at station F3 and from 0.020 ± 0.012 to 0.15 ± 0.076 C_egg_ C_female_^−1^ day^−1^ at station CB (Fig. 8a). The seasonal patterns were bimodal, with winter (January or February) and spring (May) peaks. The spring peaks were slightly higher than winter peaks. The SEPR for subitaneous eggs (subitaneous SEPR) also showed a bimodal pattern but was higher in January or February in both years (0.052–0.092 C_egg_ C_female_^−1^ day^−1^), except for station CB in 2017 (Fig. 8b).

When subitaneous SEPR (Fig. 7b) was compared with total EPR (Fig. 8b), the difference in subitaneous SEPR between summer and winter was smaller than that in total EPR. This finding is explained by body size; the body size of the winter population was larger; therefore, egg production per unit of carbon was lower in winter. The SEPR for unhatched eggs (unhatched SEPR), including resting eggs, peaked in early May to early June at 0.044–0.11 C_egg_ C_female_^−1^ day^−1^).

### Seasonal changes in PEPR

**Figure 9:**
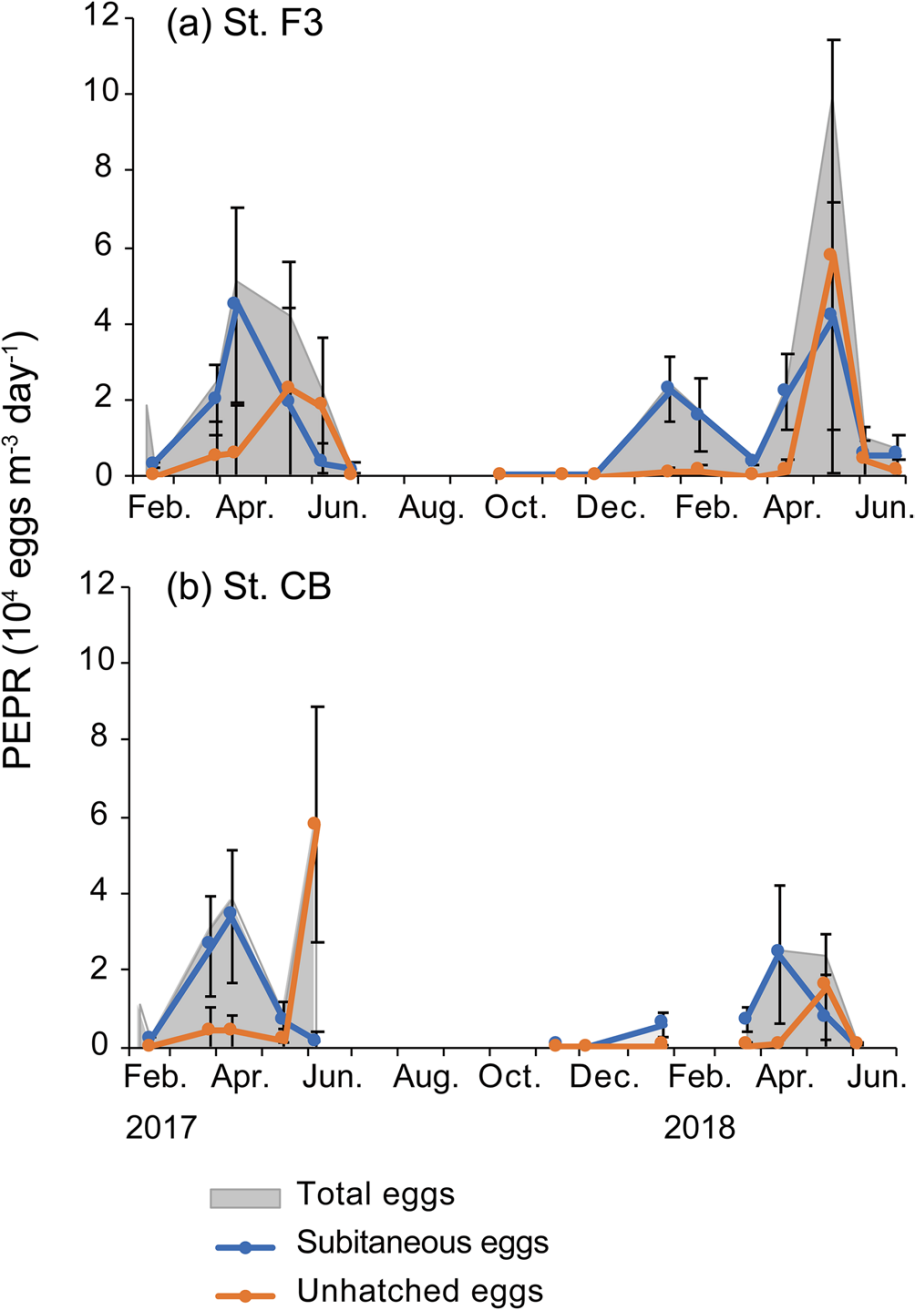
Seasonal changes in population egg production rate (PEPR) of *Acartia omorii* at St. F3 (**a**) and St. CB (**b**) from February 2017 through June 2018.

Total PEPR was quite low (< 0.5 × 10^4^ eggs m^−3^ day^−1^) in February 2017 at both stations (Fig. 9) due to low abundance (Fig. 3). With increases in population density (Fig. 3) and the proportion of males (Fig. 6), PEPR exceeded 3 × 10^4^ eggs m^−3^ day^−1^ in March or April at both stations (Figs. 7, 9). From April, PEPR decreased to nearly 0 eggs m^−3^ day^−1^ in July at station F3. At station CB, unhatched PEPR increased to 5.9 × 10^4^ eggs m^−3^ day^−1^. In 2018, two peaks of total PEPR were detected at both stations. The first peak in January or February was mostly due to subitaneous eggs. Overall, high subitaneous egg production occurred simultaneously with high population densities or high proportions of females.

The second peak of total PEPR was due to increase in unhatched PEPR at both stations. The highest values were recorded in May at both stations (5.77 × 10^4^ and 1.58 × 10^4^ eggs m^−3^ day^−1^ at stations F3 and CB, respectively), exceeding subitaneous PEPR. In late June, when unhatched PEPR was higher than subitaneous PEPR, the population density drastically decreased by 78–97.9% from the previous month (Fig. 3).

### Seasonal changes in egg type composition

**Figure 10:**
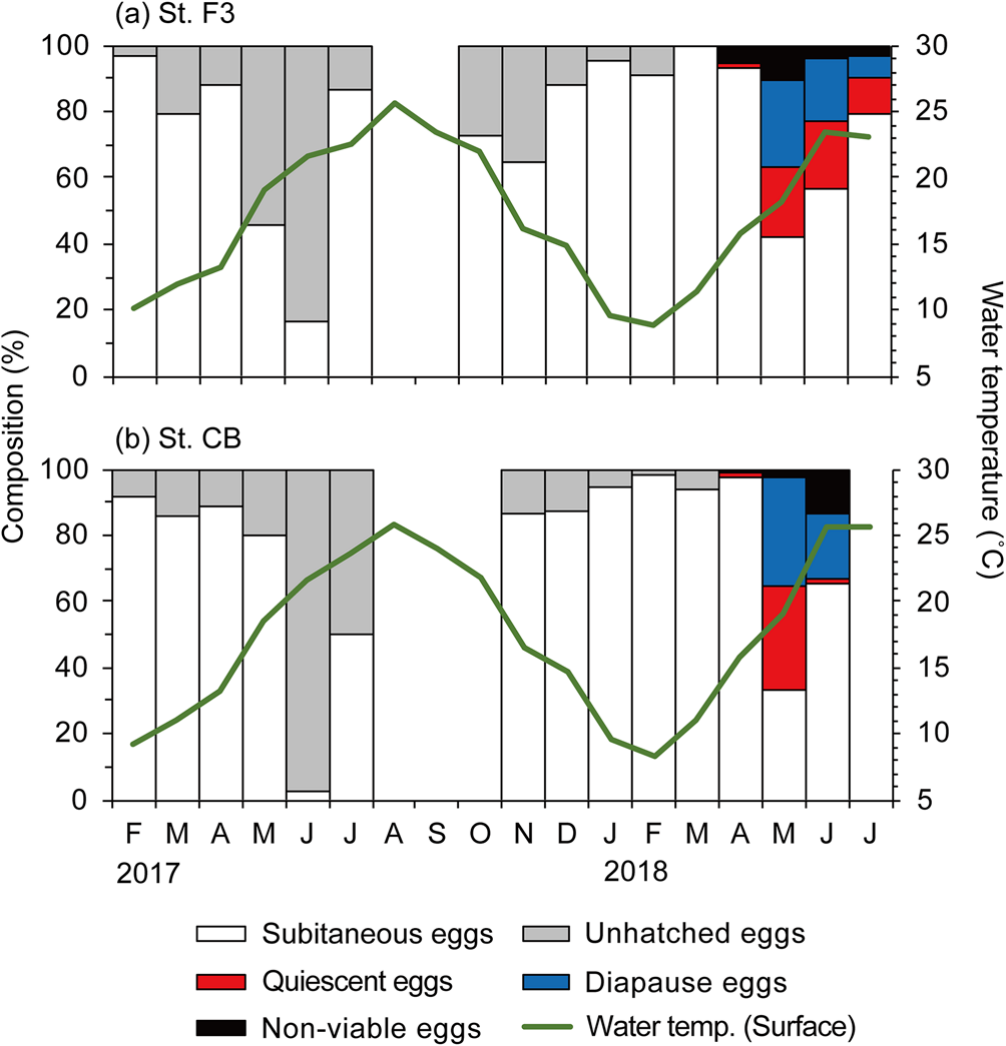
Seasonal changes in the composition of egg type in total produced eggs by *Acartia omorii* at St. F3 (**a**) and St. CB (**b**) from February 2017 through June 2018. Lines in both panels indicate surface water temperature.The non-viable, diapause and quiescent eggs were only investigated from April to June 2018.

The proportion of unhatched eggs to total eggs from February to April in 2017 was low (< 20.7%) (Fig. 10). It increased to 54.3% at station F3 on May 16, when the water temperature reached 19 °C. The proportion of unhatched eggs to total eggs reached a maximum (83.7% at station F3 and 97.6% at station CB) on June 7, when the water temperature was 21 °C at both stations. On June 28, when the water temperature exceeded 22 °C, the proportion of unhatched eggs decreased to 13.6% and 50% at stations F3 and CB, respectively. The proportion remained low (11.8–35.1%) from October to December at the two stations. Similarly, in 2018, it remained low (< 8.9%) from January to April. It peaked on May 14 (58.1% at station F3 and 64.9% at station CB). Thereafter, it decreased to 31.3% at station CB on June 4 and to 20.3% at station F3 on June 26.

In 2018, we further investigated the proportion of unhatched eggs. From April to June, the proportion of quiescent eggs was negligible, ranging from 1.7% to 1.9% at the two stations in April. It became high (> 20.0%) at station F3 in May and June and peaked at 31.3% at station CB in May. Diapause eggs were undetected in April. Diapause eggs appeared in May, and the proportion of diapause eggs to total eggs peaked at 26.5% at station F3 and 32.8% at station CB and remained high on June 4 (18.9% at station F3 and 19.7% at station CB). Non-viable eggs accounted for 0.9–13% of total egg production from March to June in 2018.

### Changes in egg types produced by individual females

**Figure 11:**
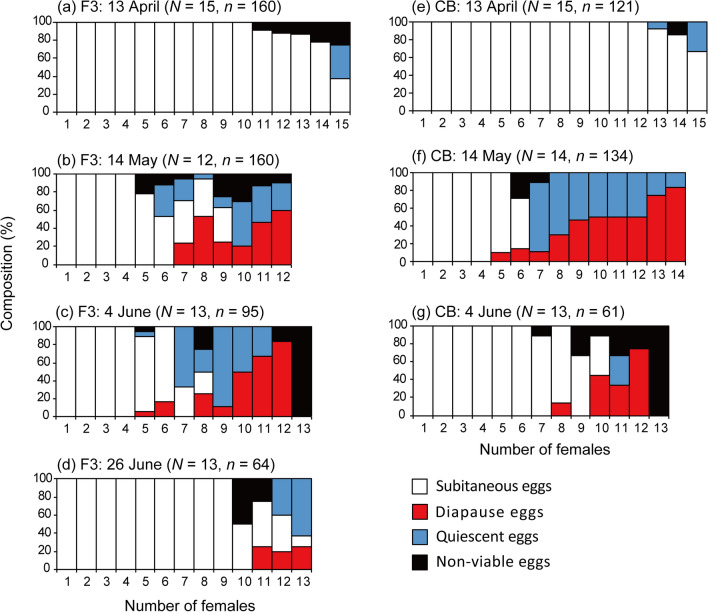
Temporal variation in the composition of each egg type in total produced eggs produced by individual females of *Acartia omorii* from April 13 to June 26, 2018 at St F3 (**a**–**d**), April 13 to June 4, 2018 at St. CB (**e**–**g**). *N*: the number of females used in each experiment *n*: the number of eggs used in each experiment.

On April 13, all 30 females produced subitaneous eggs and 3 females produced quiescent eggs (Fig. 11). On May 14, some females simultaneously produced quiescent and diapause eggs. Six of 12 females at station F3 and 8 of 14 females at station CB produced both quiescent and diapause eggs. Females also simultaneously produced quiescent and diapause eggs in June, although the proportion of females producing both quiescent and diapause eggs decreased on June 4 at station CB and on June 26 at station F3. This decrease coincided with a sharp decrease in abundance (Fig. 3). On June 4 at station CB and on June 26 at station F3, 8 and 10 of 13 females produced subitaneous eggs, respectively.

### Seasonal variation in phytoplankton

**Figure 12:**
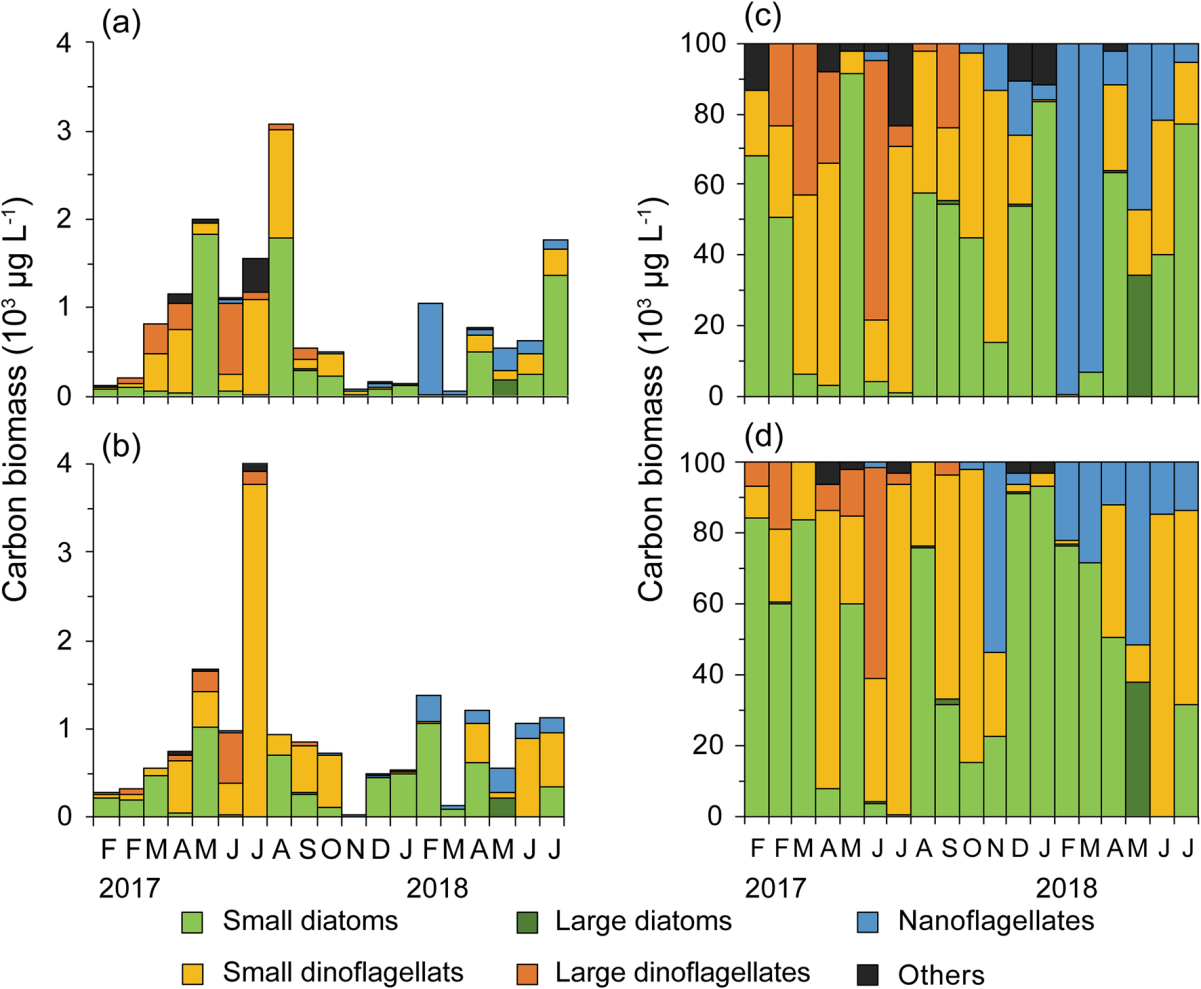
Seasonal variation in phytoplankton carbon biomass at St. F3 (**a**), St. CB (**b**) and composition of carbon biomass at St. F3 (**c**), St. CB (**d**) from February 6, 2017 through June 26, 2018. In 2017, the first F is on February 6, the second F is on February 14.

In 2017, the total phytoplankton carbon biomass increased from February to April at both stations (Figs. 12a,b). Relatively high values (0.6–4.0 × 10^3^ µg C L^−1^) were recorded between May and August when subitaneous SEPR was relatively high (ca. 0.05 C_egg_ C_female_^−1^ day^−1^). Edible phytoplankton (small diatoms and small dinoflagellates) accounted for > 50% of the phytoplankton from February to May. The dominant taxa during this period were *Cerataulina pelagica*, genus *Skeletonema*, *Thalassiosira* spp., and *Cheatoceros* spp. When the proportion of subitaneous SEPR decreased and was compensated by unhatched SEPR in June, the carbon biomass of large dinoflagellates, mainly *Ceratium furca*, tentatively increased at both stations.

From December 2017 to February 2018, the phytoplankton carbon biomass was low at station F3. However, most phytoplankton comprised small diatoms and nanoflagellates at both stations. When subitaneous SEPR was high in January, small diatoms accounted for > 83% of total phytoplankton carbon biomass. At that time, *Skeletonema* was predominant, with 63–85% of total phytoplankton carbon biomass. In May, when unhatched SEPR peaked at both stations (0.062–0.068 C_egg_ C_female_^−1^ day^−1^), large diatoms of *Pleurosigma* spp. accounted for 35–38% of total phytoplankton carbon biomass.

### Multiple regression analysis

The optimal multiple regression equations for subitaneous SEPR and unhatched SEPR were derived from the standardized data to analyze the relationship between EPR and environmental factors:

- Subitaneous SEPR=0.29 DaS−0.28 T+0.15 DfS (*n*=350, *r*=0.40, *p*<0.02),
- Unhatched SEPR = 0.32 DfL + 0.32 DaL + 0.20 T (*n*=350, *r*=0.50, *p*<0.01),

where DaS is the proportion of small diatoms to total phytoplankton carbon biomass, DfS is the proportion of small dinoflagellates, DaL is the proportion of large diatoms, DfL is the proportion of large dinoflagellates, and T is the bottom water temperature.

Subitaneous SEPR positively correlated with small diatoms and small dinoflagellates and negatively correlated with bottom water temperature. Unhatched SEPR positively correlated with large diatoms, large dinoflagellates, and water temperature. Therefore, the effect of water temperature on egg production differed according to egg type. The negative relationship between unhatched SEPR and large phytoplankton proportion (> 100 µm) suggests that the presence of inedible phytoplankton stimulates unhatched egg production.

## Discussion

### Population dynamics

The abundance of *A. omorii* peaked in April 2016, March 2017, and April–May 2018, ranging from 2.99 × 10^4^ to 6.73 × 10^4^ individuals m^−3^ (Fig. 3, Table 1). Anakubo and Murano^28^ reported that the abundance of *A. omorii*, including individuals from the nauplius to adult stage, peaked at 5.34 × 10^4^ m^−3^ in April 1981 in Tokyo Bay. Itoh and Aoki^18^ surveyed *A. omorii* in Tokyo Bay from March 1990 to November 1992 and found that abundance peaked at 2.13 × 10^4^ individuals m^−3^ in March. Tachibana et al.^40^ reported that abundance peaked at 2.60 × 10^4^ individuals m^−3^ in April in 2007. The peak abundance in Tokyo Bay apparently occurs between March and May; this pattern was also observed in the Seto Inland Sea (Table 1). According to Kasahara et al.^15^, the peak period of abundance of adults and later-stage copepodites in the Seto Inland Sea occurred in mid-May at > 3.5 × 10^4^ individuals m^−3^. The abundance of adults and later-stage copepodites significantly decreased in July. The planktonic population disappeared from the water column in mid-August and recovered in November, when the water temperature was < 20 °C. Similarly, Liang and Uye^17^ reported that the abundance of *A. omorii*, including nauplii, in the Seto Inland Sea increased in February 1987 to 5.8 × 10^4^ individuals m^−3^; a second peak was observed in June at 6.9 × 10^4^ individuals m^−3^. The planktonic population disappeared in late July and appeared again in late October. Therefore, the peak of the abundance of this species (copepodites and adults) in Tokyo Bay was close to that in the Seto Inland Sea. Additionally, the period of disappearance of the planktonic population (late July or August–October) was almost the same in the Seto Inland Sea and Tokyo Bay^16,18,28,40^. The water temperature in both Tokyo Bay and the Seto Inland Sea ranged from 8 °C to 28 °C. The seasonal dynamics of *A. omorii* populations are probably common in warm inner bays where summer water temperatures exceed 25 °C.

**Table 1:** Abundance of *Acartia omorii* and water temperature range in each site in Japan.

Site	Temperature (°C)	Max abundance (ind. m^−3^)	Peak period	Low abundance period	Year	References
Otsuchi Bay	3.6 (Mar.)–22.4 (Sep.)	about 3 × 10^3^ (adults)	September (2011)	*Sep.–Jan. (2012)	2011–2013	Nishibe et al.^41^
Okirai Bay	4.4 (Mar.)–20.3 (Nov.)	2.8 × 10^2^	April	*Oct.–Nov	2007–2009	Yamada et al.^42^
Onagawa Bay	4.7 (Feb.)–23.2 (Sep.)	about 4 × 10^3^ (adults)	September	Dec.–May	1976–1978	Uye^14,38^
Seto Inland Sea	7.5 (Feb.)–27.8 (Aug.)	about 3.5 × 10^4^ (adult and late copepodites)	mid May	*Aug.–Oct	1973–1974	Kasahara et al.^15^
	8.9 (Feb.)–28.2 (Sep.)	5.8 × 10^4^, 6.9 × 10^4^ (nauplius–adult)	February, June	*late July–Oct	1986–1987	Liang and Uye ^17^
Tokyo Bay	8.1 (Feb.)–26.5 (Aug.)	5.3 × 10^4^ (nauplius–adult)	April	July–Jan	1980–1982	Anakubo and Murano^28^
	–	1.1 × 10^4^ (adult and copepodites)	March	Aug.–Oct	1981–1988	Nomura and Murano^16^
	9.9 (Feb.)–28.4 (July)	2.1 × 10^4^ (adult and copepodites)	March	July–Dec	1990–1992	Itoh and Aoki^18^
	8.6 (Feb.)–28.9 (Aug.)	about 2.6 × 10^4^ (adult and copepodites)	April	Sep.–Nov	2006–2008	Tachibana et al.^40^
	8.2 (Feb.)–27.6 (Aug.)	2.99 × 10^4^–6.7 × 10^4^ (adult and copepodites)	March–May	* Sep.–Nov	2016–2018	The present study

*Population completely disappeared in water column, the others are considerable low abundance.

In contrast, the abundance of *A. omorii* in Onagawa Bay peaked at 4 × 10^3^ individuals m^−3^ in September and decreased to < 5 × 10^2^ individuals m^−3^ in December^14,38^. The water temperature in Onagawa Bay ranged from 4.5 °C to 23.2 °C, which was 3–5 °C lower than that in Tokyo Bay and the Seto Inland Sea (Table 1). The peak population abundance period is likely to depend on the temperature range. However, this is not the case for Okirai Bay and Otsuchi Bay, where water temperature range was lower than that in Onagawa Bay, and the planktonic populations disappeared in autumn, as in Tokyo Bay and the Seto Inland Sea (Table 1). In these two bays, *A. omorii* abundance was low even at the peak, with 2.9 × 10^2^ and 3 × 10^3^ individuals m^−3^ in Okirai Bay and Otsuchi Bay, respectively. In Otsuchi Bay, *A. hudsonica* was present throughout the study and showed remarkable abundance peaks (> 1.5 × 10^4^ individuals m^−3^) in May^41^. Furthermore, in Okirai Bay, *A. steueri* appeared from July to March and peaked in September, with the maximum value exceeding 1.5 × 10^3^ individuals m^−3^^42^. In these bays, copepods are more diverse, and *A. omorii* abundance may be controlled by competition among these *Acartia* species. *A. omorii* was predominant in Tokyo Bay, the Seto Inland Sea, and Onagawa Bay^14–16,18,28,40,43^. These results suggest that the population dynamics of *A. omorii* are probably affected by competition with other copepods. When competition is at a negligible level, population dynamics may be primarily affected by water temperature via resting egg production.

It should be noted that the population dynamics is also affected by their grazers such as jellyfish. In Tokyo Bay, abundance of *Auriela aurita* s.l. was high until August with the highest abundance of 1.6 ind. M^−3^^44^. Itoh et al.^45^ found that vertical distribution of *A. omorii* was affected by *Auriela* sp. in the bay. *A. omorii* had a sharp peak at 9 m depth in the vertical profile, however the abundance was very low at 10- and 11 m depth where *Aruriela* sp. was abundant (> 50 g wet weight m^−3^). Hence summer jellyfish bloom probably has a strong negative impact on the abundance of *A. omorii*. Unfortunately, we do not have enough information on jellyfish abundance in Onagawa Bay, effect of jellyfish should be considered for the better understanding of *A. omorii* dynamics in future.

*A. omorii* abundance became zero only from September to November 2016 in the 3 years of the present study. However, in other years, *A. omorii* appeared at low abundance in summer, as reported in previous studies conducted in Tokyo Bay ^16,18,28,40^. The period of complete disappearance was short, and low densities were recorded throughout the year in most studies (Table 1), indicating that the population is maintained in the planktonic stages even under unfavorable conditions in Tokyo Bay.

### EPR and egg type dynamics

SEPR peaked in winter (January or February) and May in both years at both stations (Fig. 8a). The second peaks were caused by increase in unhatched egg production, except for station CB in 2017 (Figs. 7b,c and 8b,c). Increase in unhatched egg production occurred when the surface water temperature exceeded 18 °C (Fig. 2a) and day length increased to > 14 h, similar to that reported by Uye^5^. From January to May 1982, 80% of eggs that were newly spawned by *A. omorii* females collected in Fukuyama Harbor of the Seto Inland Sea were subitaneous; resting eggs appeared in June when the surface water temperature exceeded 17.5 °C. In the present study, on June 9, 32% of the total eggs produced were resting and did not hatch within 14 days but hatched after being reincubated at 15 °C for 2 weeks. Uye^5^ also demonstrated the effect of photoperiod on egg production by *A. omorii* via laboratory experiments under two temperature conditions (15 °C and 20 °C ± 1 °C). Approximately half were resting eggs under l4L–10D photoperiodic conditions at both water temperatures, indicating that photoperiod is important for shift to resting eggs. Many copepods have dormancy strategies at the thermal limits of species distribution^46^. In Tokyo Bay, the photoperiod exceeds 14 h in mid-May, when the water temperature usually exceeds 18 °C (Fig. 2a). Therefore, higher water temperatures and increased day length periods are synchronized in early summer in Tokyo Bay and may serve as cues for diapause egg production.

Unhatched PEPR exceeded subitaneous PEPR from May to June (Fig. 9), when abundance drastically decreased to < 1 × 10^4^ individuals m^−3^ (Fig. 4). Itoh and Aoki^18^ reported that resting eggs of *A. omorii* were abundant in the bottom sediment of Tokyo Bay in June, when the water temperature increased to approximately 20 °C. In the present study, in May 2018, 37% (station F3) and 47% (station CB) of unhatched eggs were quiescent and hatched immediately when the water temperature decreased (Fig. 10). Therefore, this unhatched PEPR probably avoided an unsuitable temperature for hatching and development. Similar results were reported for *A. steueri*; the PEPR for diapause eggs accounted for up to 98% of total PEPR from May to June^10^. Subsequently, adult abundance became low until September or October. The production rate of resting eggs reaches its highest level in common with these species just before the warm period when the population abundance becomes low. These *Acartia* species may have a strategy to avoid high temperatures by sensing increase in water temperature and producing diapause eggs with a long dormant period just before summer.

The egg types produced by *A. omorii* differed among females (Fig. 11). Even on the same day, many females produced only subitaneous eggs or only resting eggs (quiescent and diapause eggs). This difference may be related to the age. Walton^47^ reported that *Onychodiaptomus birgei* females initially produced subitaneous eggs and then switched to producing resting eggs during their lifetimes. The difference may also be related to the history of environmental conditions each individual experiences in the field according to their age. In contrast, few females simultaneously produced all egg types: subitaneous, quiescent, and diapause eggs (Fig. 11). Other *Acartia* species also simultaneously produce subitaneous and resting eggs^4,37,48^. For example, Drillet et al.^37^ found that during 2 days of incubation, half of the females of *A. tonsa* simultaneously produced subitaneous and delayed-hatching eggs. Takayama and Toda^4^ also reported that several females of *A. japonica* simultaneously produced subitaneous, diapause, and delayed-hatching eggs. The simultaneous production of different eggs types by an individual is probably related to environmental factors in a complex way and therefore should be investigated using molecular and endocrine approaches.

In the present study, most females produced only subitaneous eggs in the early period of *A. omorii* appearance from November to April (Fig. 9); the proportion of females producing both diapause and quiescent eggs was high (> 45%) (Fig. 6) in the late period of *A. omorii* appearance (in May at station CB and in early June at station F3). However, in early June at station CB and late June at station F3, when the abundance sharply decreased, the proportion of females producing subitaneous eggs increased, whereas those producing quiescent eggs decreased from the previous month (Fig. 11). Uye^5^ reported a similar result; the proportion of diapause eggs to total eggs produced by *A. omorii* in the Seto Inland Sea peaked on June 9 and then reduced by half by June 30. Quiescent eggs have been defined as subitaneous eggs with arrested development that remain in a quiescent stage in unsuitable environmental conditions^49^. Uye^5^ defined diapause eggs as eggs that did not hatch during 2 weeks at in situ water temperatures but hatched within 2 weeks when the incubation temperature decreased to 15 °C. These eggs may be classified as quiescent eggs in the present study. The results of Uye^5^ and the present study suggest that not all produced eggs of this species shift from subitaneous to quiescent eggs at higher water temperatures.

As mentioned in the previous subsection, a planktonic population of *A. omorii* has been found in mid-summer at low abundance in Tokyo Bay^16,18,28,40^ (Fig. 3). Similar results were reported in Maizuru Bay^50^. Itoh et al.^45^ investigated the vertical distribution of copepods at 1-m depth intervals at station F3 in Tokyo Bay in mid-summer, when hypoxia develops near the bottom, and showed that *A. omorii* population had a sharp peak, with densities exceeding 1.5 × 10^3^ individuals m^−3^, at 8 m at a water temperature of 18 °C and just above the hypoxic zone^45^. This suggests that *A. omorii* maintains a planktonic stage even at low density in mid-summer, whereas most of the population estivates by forming resting eggs in bottom sediments. These mid-summer populations are presumably hatched from subitaneous eggs spawned in mid-July (Figs. 7, 10). Uye^5^ also reported that more than half of the eggs were still subitaneous in late July in the Seto Inland Sea. Therefore, we tentatively think that this phenomenon is a bet-hedging strategy of *A. omorii* in an unfavorable and uncertain environment. In contrast, Ueda^50^ stated that the increase in subitaneous EPR in summer was due to immature development of this species. Thus, these remaining populations may not contribute to the autumnal development of the population. To understand how *A. omorii* survive in mid-summer, more detailed field investigations are warranted, including egg and nauplii dynamics in the water column, egg hatching process from sediments, and differences in endogenous factors in individual females producing subitaneous and diapause eggs in summer.

Information on *A. omorii*’s delayed-hatching eggs is strictly limited. Delayed-hatching eggs are eggs hatching over a wide time span regardless of environmental conditions^4,11^. Takayama and Toda^4^ defined the unhatched eggs of *A. japonica* hatching during 72 h–50 days as “delayed-hatching eggs.” Thus, delayed-hatching eggs may have been included in the quiescent and diapause eggs defined in this study. Our results showed that no eggs hatched between 48–96 h and 7 days in the experiment at in situ water temperature; many quiescent eggs hatched within a few days after reincubation at lower water temperatures (Figs. 10, 11). Therefore, delayed-hatching eggs may not have been produced in the present study.

### Effects of water temperature on the production of subitaneous and diapause eggs

Multiple regression analysis revealed that subitaneous SEPR negatively correlated with bottom water temperature, inconsistent with the results of Uye^3^. EPR and copepod growth generally increase with increased water temperature^51^. Uye^3^ reported that EPR of *A. omorii* also increased with water temperature; they developed a simple model equation describing the fecundity of *A. omorii* in Onagawa Bay via a laboratory experiment:

$${\text{F}} = 0.000{331 }\left( {{\text{T}} + {12}.0} \right)^{{{3}.{25}}} {\text{SW}}_{{\text{f}}} /\left( {0.{47}0 + {\text{S}}} \right),$$

where F is daily fecundity (eggs female^−1^ day^−1^), T is water temperature (°C), S is chlorophyll *a* concentration (µg L^−1^), and W_f_ is female carbon content (µg). The fecundity predicted by the above described model was similar to the observed EPR of this species in Onagawa Bay^3^.

Many studies have used Uye’s equation to estimate *A. omorii* egg production. Kang et al.^52^ reported *A. omorii*’s EPR in Ilkwang Bay to be 22–57 eggs female^−1^ day^−1^, which was higher than that in the present study (1.6–18.7 eggs female^−1^ day^−1^) (Fig. 7a). Liang and Uye^17^ estimated *A. omorii*’s EPR in the Seto Inland Sea by two methods: the above described model (estimated incubation fecundity)^3^ and based on the number of eggs remaining in the water column and the adult female population (egg-ratio fecundity)^53^. In the Seto Inland Sea, the estimated incubation fecundity was 26–60 eggs female^−1^ day^−1^ and the egg-ratio fecundity was 0.5–25 eggs female^−1^ day^−1^; the estimated incubation fecundity was always greater than the egg-ratio fecundity^17^.

Suspension-feeding copepods may ingest their own eggs and nauplii. In the Seto Inland Sea, possible egg predators were the dominant copepods *Centropages abdominalis* and *A. omorii*^54^. Based on their abundance (0.2–39 predators L^−1^) and assuming a predator clearance rate of 50 mL d^−1^, *C. abdominalis* and *A. omorii* could remove 1–86% of eggs in the water column per day. Liang and Uye^17^ noted that their predators were abundant when the abundance of surviving eggs in the water column was low; therefore, they tentatively concluded that the difference between the two estimates was due to egg loss by predation, including cannibalism. However, it is unlikely that fecundity reached its highest value (> 50 eggs female^−1^ day^−1^) in mid-July when the population disappeared from the water column^17^. At that time, the water temperature was 25 °C, which also does not support the increase in fecundity observed by Liang and Uye^17^.

The model equation of Uye^3^ was derived from Onagawa Bay, where the average water temperature is 7.7–21.9°C^55^. In laboratory experiments using *A. omorii* from Onagawa Bay, EPR decreased when the water temperature exceeded 22.5°C^3^. In Uye’s equation^3^, the decrease in egg production above 22.5 °C was not foreseen, whereas water temperature exceeded 22.5 °C in the Seto Inland Sea^17^, Ikkwang Bay^52^, and Tokyo Bay (Fig. 2). Thus, Uye’s model equation^3^ is not applicable to these warm environments.

Based on the temperature regime, seasonal population dynamics and egg types produced are divided into two types: no resting egg production in the colder Onagawa Bay and resting egg production in the warmer Tokyo Bay and Seto Inland Sea. As mentioned above, *A. omorii* in Onagawa Bay exists throughout the year, even in summer^14^ and hardly produces diapause eggs^5,7^. However, the population almost disappears in late summer in Tokyo Bay^16,18,28,40^ and the Seto Inland Sea^15,17^. Furthermore, in these warm coastal waters, *A. omorii* produced diapause eggs just before copepodite disappearance from the water column. Therefore, a separate equation for estimating egg production should be developed, depending on the temperature regime of the habitat.

Recent climate change, particularly global warming, may affect *A. omorii*’s egg production. In Tokyo Bay, between 1955 and 2015, the water temperature increased by 1.0 °C and 0.94 °C at the surface and bottom layers, respectively, in winter and autumn^56,57^. In summer, the water temperature at both the surface and bottom layers decreased, probably due to strengthened estuary circulation^56,57^. Considering the response of *A. omorii* to water temperature, the increase in winter temperature might reduce subitaneous egg production, resulting in delayed population increase. In contrast, the decrease in summer temperature might lead to reduced diapause egg production per amount of body carbon. The long-term trends of water temperature might have different effects on each egg type production and alter the dynamics of *A. omorii* egg production.

### Effects of phytoplankton composition on the production of subitaneous and diapause eggs

The EPR of *A. omorii* may be saturated at low (1–2 µg L^−1^) chlorophyll *a* concentrations^3,19^. However, multiple regression analysis revealed that small diatoms stimulate subitaneous SEPR (Figs. 8, 12). The EPR at station CB was quite high (> 14 eggs female^−1^ day^−1^) in January and February 2018, when the diatoms comprised *Skeletonema* and *Chaetoceros*. In contrast, at station F3, EPR drastically decreased from 18.7 ± 6.3 eggs female^−1^ day^−1^ in January to 8.4 ± 4.6 eggs female^−1^ day^−1^ in February 2018. The EPR at station F3 in February was significantly lower than that at station CB (Tukey's post hoc test, *p* < 0.01). In this month, small-sized nanoflagellates accounted for 99% of total phytoplankton carbon biomass. Studies have reported that diatoms have a positive effect on copepod EPRs in both laboratory experiments^58,59^ and field investigations^60–63^. In the present study, hatching success was also high (> 95%) at station CB in January and February 2018, suggesting that small diatoms ingestion enhances *A. omorii*’s egg production.

It is also likely that small nanoflagellates have an adverse effect on egg production. At station F3, the proportion of nanoflagellates to total phytoplankton carbon biomass was high (> 93%) in February and March (Fig. 12), when EPR was quite low (< 8.4 eggs female^−1^ day^−1^) (Fig. 7a). The EPR of *Temora longicormis* was reduced from 20 eggs female^−1^ day^−1^ when they were feeding on *Thalassiosira weissflogii* to 10 eggs female^−1^ day^−1^ when they were feeding on *Tetraselmis suecica* and to < 5 eggs female^−1^ day^−1^ when *Dunaliella tetriolecta* was used as prey^64^. Sopanen et al.^65^ reported that the haptophyte *Prymnesium* had a strong negative effect on the EPR of *A. clausi*, even when it was fed in a mixture with *Rhodomonas*, which is known to support reproduction in *Acartia*^66^. The bloom of some nanoflagellate species may reduce copepod egg production.

Unhatched SEPR positively correlated with the proportion of large diatoms to total phytoplankton carbon biomass and proportion of dinoflagellates to total phytoplankton carbon biomass (Figs. 8, 12). Large diatom biomass was mostly accounted for by the genus *Pleurosigma* (cell size 160 µm), and the most common large dinoflagellates were *Ceratium furca* (cell size 145 µm) and *C. fusus* (cell size 350 µm). These sizes are probably above or close to the maximum size of the edible prey for *A. omorii*^36^. Therefore, both large diatoms and dinoflagellates cannot be consumed by *A. omorii*. Furthermore, in early June 2017 and May 2018, when unhatched eggs accounted for the highest proportion of total eggs produced during each year (Fig. 10), the proportion of small diatoms was quite low (< 4%), while that of large phytoplankton carbon biomass was high (> 60% and > 35% in early June 2017 and May 2018, respectively) (Fig. 12). Food availability may be involved in egg type switching^37^. The fraction of delayed-hatching eggs of *A. tonsa* was greater at low-food concentrations (*Rhodomonas salina*, 2000 cells mL^−1^) than at high-food concentrations (40,000 cells mL^−1^)^37^. The primary cues for switching from subitaneous eggs to diapause eggs in Tokyo Bay seem to be water temperature and photoperiod. However, low-food availability, such as during the temporary disappearance of small diatoms and the bloom of inedible species, might stimulate diapause egg production by *A. omorii*.

Finally, the possible effect of prey availability on the small-scale horizontal distribution of *A. omorii* in Tokyo Bay should be noted. Itoh and Nishida^67^ reported that the abundance of *A. omorii* was higher on the east side of Tokyo Bay than on the west side. Chlorophyll *a* concentration was generally higher on the west side of Tokyo Bay in summer; however, on the east side of Tokyo Bay, it was higher in winter^68^. Even in the present study, diatom blooms were observed every winter in the vicinity of station CB, whereas diatom biomass was relatively low in the vicinity of station F3 every year (Fig. 12). Therefore, the horizontal distribution of phytoplankton may also influence *A. omorii* abundance through egg production, which is positively affected by small diatoms.

Our preliminary results show that edible diatoms may stimulate subitaneous EPR; however, the effects of individual diatom species are currently unknown. The production of cytotoxic compounds, including polyunsaturated aldehydes, by diatoms may vary at the genus, species or clonal level^69,70^. Toxicity tolerance also varies with copepod species^71,72^. Therefore, to clarify the effect of diatom species on *A. omorii* egg production, in situ monitoring of cytotoxic compounds and chemical and molecular analysis of the toxicity tolerance of *A. omorii* is warranted.

## Conclusions

We showed that *A. omorii* produces resting eggs just before the disappearance period in summer when the surface water temperature exceeds 18 °C. Subitaneous egg production by *A. omorii* was highest in winter and negatively correlated with surface water temperature. Subitaneous egg production was enhanced by small diatoms. Water temperature and photoperiod may be important cues for switching to diapause egg production; however, food availability may also be involved. The phytoplankton composition in the food environment, in addition to abiotic factors, should be taken into account for accurate prediction of the population dynamics of the dominant copepod *A. omorii*.

## Supplementary Information

Supplementary Information 1.

Supplementary Information 2.

Supplementary Information 3.

Supplementary Information 4.

## Data Availability

All data generated or analysed during this study are included in this published article;
